# *HRS1* Acts as a Negative Regulator of Abscisic Acid Signaling to Promote Timely Germination of *Arabidopsis* Seeds

**DOI:** 10.1371/journal.pone.0035764

**Published:** 2012-04-24

**Authors:** Chongming Wu, Juanjuan Feng, Ran Wang, Hong Liu, Huixia Yang, Pedro L. Rodriguez, Huanju Qin, Xin Liu, Daowen Wang

**Affiliations:** 1 The State Key Laboratory of Plant Cell and Chromosome Engineering, Institute of Genetics and Developmental Biology, Chinese Academy of Sciences, Beijing, China; 2 Graduate University of Chinese Academy of Sciences, Beijing, China; 3 Instituto de Biología Molecular y Celular de Plantas, Universidad Politécnica de Valencia-Consejo Superior de Investigaciones Científicas, Valencia, Spain; Iwate University, Japan

## Abstract

In this work, we conducted functional analysis of *Arabidopsis HRS1* gene in order to provide new insights into the mechanisms governing seed germination. Compared with wild type (WT) control, *HRS1* knockout mutant (*hrs1-1*) exhibited significant germination delays on either normal medium or those supplemented with abscisic acid (ABA) or sodium chloride (NaCl), with the magnitude of the delay being substantially larger on the latter media. The hypersensitivity of *hrs1-1* germination to ABA and NaCl required *ABI3*, *ABI4* and *ABI5*, and was aggravated in the double mutant *hrs1-1abi1-2* and triple mutant *hrs1-1hab1-1abi1-2*, indicating that *HRS1* acts as a negative regulator of ABA signaling during seed germination. Consistent with this notion, *HRS1* expression was found in the embryo axis, and was regulated both temporally and spatially, during seed germination. Further analysis showed that the delay of *hrs1-1* germination under normal conditions was associated with reduction in the elongation of the cells located in the lower hypocotyl (LH) and transition zone (TZ) of embryo axis. Interestingly, the germination rate of *hrs1-1* was more severely reduced by the inhibitor of cell elongation, and more significantly decreased by the suppressors of plasmalemma H^+^-ATPase activity, than that of WT control. The plasmalemma H^+^-ATPase activity in the germinating seeds of *hrs1-1* was substantially lower than that exhibited by WT control, and fusicoccin, an activator of this pump, corrected the transient germination delay of *hrs1-1*. Together, our data suggest that *HRS1* may be needed for suppressing ABA signaling in germinating embryo axis, which promotes the timely germination of *Arabidopsis* seeds probably by facilitating the proper function of plasmalemma H^+^-ATPase and the efficient elongation of LH and TZ cells.

## Introduction

Seed germination marks the beginning of a new growth cycle in higher plants, and is thus subject to complex controls by both internal and environmental cues [1], [2]. Over the last few years, considerable efforts have been devoted for studying the molecular genetic basis of seed germination. Using *Arabidopsis thaliana* as a model and molecular genetic approaches, a variety of genes functioning during seed germination have been identified [2]–[4]. Many of these genes encode enzymes involved in hormone biosynthesis or catabolism, or components acting in the signal transduction chains of one or more hormones [4]–[8]. Owing to the fundamental importance of abscisic acid (ABA) in controlling plant growth and development in the presence of environmental stresses, the genes that take part in ABA biosynthesis, turnover or signal transduction are frequently found involved in the control of seed germination [4]–[6], [9]–[11]. In general, functional deficiencies of the positive regulators of ABA response tend to stimulate seed germination, whereas debilitating mutations of the negative regulators of ABA response are inclined to inhibit seed germination [3], [4], [12]–[14]. Collectively, the available molecular genetic data reinforce the repressive function of ABA in seed germination proposed in past physiological studies. When germination begins under favorable conditions, the repression mediated by ABA is decreased rapidly because of reductions in both ABA content and the functionality of ABA signaling components [8]. During germination, ABA content must be kept low, and ABA signaling must be actively suppressed, because artificial supply of ABA to the growth medium, or mutation of the key negative regulators of ABA signaling (such as ABI1 and several structurally related type 2C protein phosphatases), can lead to significant delays in germination [15]–[20]. Interestingly, immediately after germination (i.e., after radicle emergence), ABA may inhibit further growth of the young seedling root if abiotic stress is encountered in the environment. This postgermination arrest, requiring a functional ABA signal transduction chain, is likely to be an adaptive response of newly germinated seedlings to harmful environments [21]–[23].

Despite the progress described above, our understanding of the complex mechanisms governing seed germination remains fragmented, especially with respect to relationships among the genes regulating seed germination, the hormone signaling pathways active in germinating seeds, and the cell growth and physiological events critical for timely germination in normal or abiotic stress environments. In *Arabidopsis*, seed germination is a tightly coordinated process involving radicle growth and testa and endosperm rupture [1], [24], [25], and a meticulous study has demonstrated that radicle emergence, the hallmark of germination completion, is primarily associated with cell elongation in the lower hypocotyl (LH) and the hypocotyl-radicle transition zone (TZ) of the embryo axis [26]. Although substantial progress has been made in elucidating the molecular genetic and physiological mechanisms of testa and endosperm rupture [24], [25], those regulating the elongation of LH and TZ cells prior to radicle emergence in the germinating seeds are still poorly understood. Past investigations have frequently observed the involvement of plasmalemma H^+^-ATPase in the elongation of embryo axis and germination completion, and the inhibition of embryo axis elongation and seed germination by ABA through reducing plasmalemma H^+^-ATPase activity [27]–[29]. But again the molecular genetic and cell biological mechanisms behind these physiological findings are still unclear. Although several genes, i.e., *RACK1* (encoding the receptor for activated C kinase 1), *MFT* (specifying a phosphatidylethanolamine-binding protein) and *AZF2* (coding for *Arabidopsis* zinc-finger protein 2), have recently been found to act as negative regulators of ABA signaling during *Arabidopsis* seed germination, and their expression was all detected in the germinating embryo [30]–[32], it is not known if these genes may affect cell elongation in LH and TZ and/or plasmalemma H^+^-ATPase activity in germinating embryo axis during their function in seed germination.

*HRS1* (*At1g13300*) is a member of a small gene family encoding putative G2-like transcription factors in *Arabidopsis* [33]. Its deduced protein is composed of 344 amino acid residues, and contains a conserved G2-like DNA binding domain [33]. A previous study has briefly noted that the knockout of *HRS1* leads to transient delay in *Arabidopsis* seed germination [34]. More recently, it was suggested that the N-terminus of HRS1 contains a sequence element similar to the EAR motif, and may act as a transcriptional repressor in the response of *Arabidopsis* seedlings to salt stress [35]. However, neither the function of *HRS1* in seed germination nor the possible interactions of *HRS1* with the signaling networks mediating plant stress responses (such as the ABA signaling pathway) have been well investigated in previous studies. Consequently, the objective of this work was to study in more detail the function of *HRS1* in *Arabidopsis* germination by combining molecular genetic, cell biological and physiological approaches in order to provide new insights into the mechanisms governing seed germination in higher plants.

## Results

### Germination defects of *hrs1-1*

**Figure 1:**
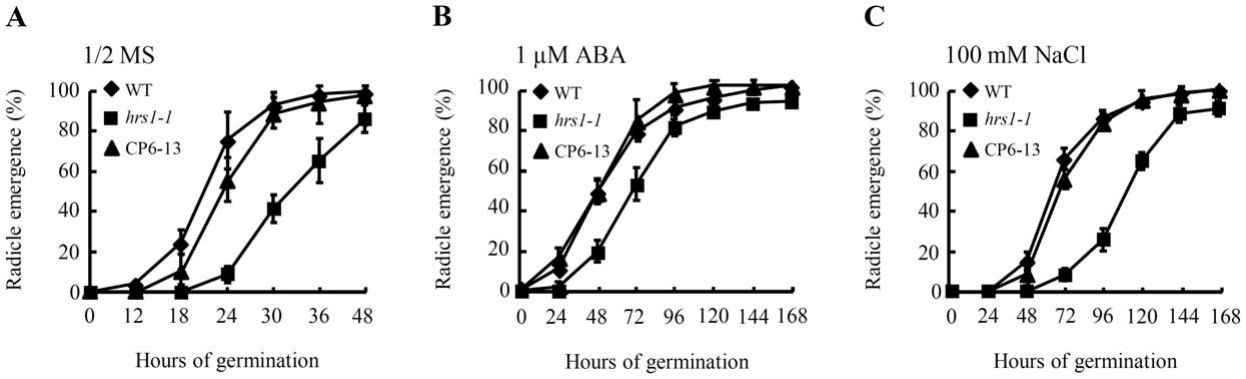
Germination defects of *HRS1* knockout mutant on different media. The seeds of wild type (WT) control, *HRS1* knockout mutant (*hrs1-1*) and complementation (CP) line (CP6-13) were stratified for 48 h at 4°C, followed by transfer to 23°C to allow germination to start on 1/2 MS medium or those supplemented with the indicated concentrations of abscisic acid (ABA) or NaCl. (A–C) The germination time courses of WT, *hrs1-1* and CP genotypes on 1/2 MS medium or those containing 1 µM ABA or 100 mM NaCl. The percentages of radicle emergence (means ± SD) at the indicated time points were each determined using the results from triplicate samples. The data shown are all typical of five separate germination experiments, and the two additional CP lines (CP19-1 and CP23-3) behaved similarly as CP6-13 during the experiments. HOG, hours of germination at 23°C.

The T-DNA insertion mutant of *HRS1*, designated *hrs1-1*, was identified previously [33]. Three independent complementation (CP) lines (CP6-13, CP19-1 and CP23-3), expressing WT *HRS1* coding sequence under *HRS1* native promoter in *hrs1-1* background, were developed in this work. The seeds of the three genotypes, i.e., WT control, *hrs1-1* and CP (represented by CP6-13), were stratified at 4°C for 48 h, followed by transfer to 23°C to allow germination to start on different media. Similar to the observation made in previous studies [24], [25], at the seed level, the germination process of WT control and *hrs1-1* began by testa rupture followed by endosperm rupture and radicle protrusion. To simplify monitoring the germination time courses of WT control and *hrs1-1*, the percentage of radicle emergence was recorded at selected time times. On 1/2 MS medium, the percentage of radicle emergence of WT control reached maximum after 48 hours of germination (HOG) at 23°C, with more than 90% of seeds germinated during the main course of the assay (from 0 to 36 HOG, Figure 1A). Compared with WT control, the percentage of radicle emergence of *hrs1-1* was significantly and consistently lower at 18, 24, 30 and 36 HOG, with the difference between the two genotypes becoming insignificant at 48 HOG (Figure 1A). By contrast, CP6-13 germinated highly similarly as WT control on 1/2 MS medium (Figure 1A). Compared to the transient defect described above, the germination of *hrs1-1* was more negatively affected in the presence of ABA or sodium chloride (NaCl) (Figure 1B and 1C). Although the germination of WT control and *hrs1-1* was both delayed on the media supplemented with 1 µM ABA or 100 mM NaCl, the scale of the delay was generally and substantially larger for *hrs1-1* than for WT control. For example, at 48 HOG, the mean percentages of radicle emergence of WT control and *hrs1-1* on 1/2 MS medium were 98% and 86%, respectively (Figure 1A), whereas at the same time point but on ABA containing medium, the corresponding values for the two genotypes were about 48% and 20%, respectively (Figure 1B). At 48 HOG and on NaCl medium, the mean percentages of radicle emergence of WT control and *hrs1-1* were around 16% and 1%, respectively (Figure 1C). The germination time course of CP6-13 resembled highly that of WT control on the media with exogenous ABA or NaCl (Figure 1B and 1C).

**Figure 2:**
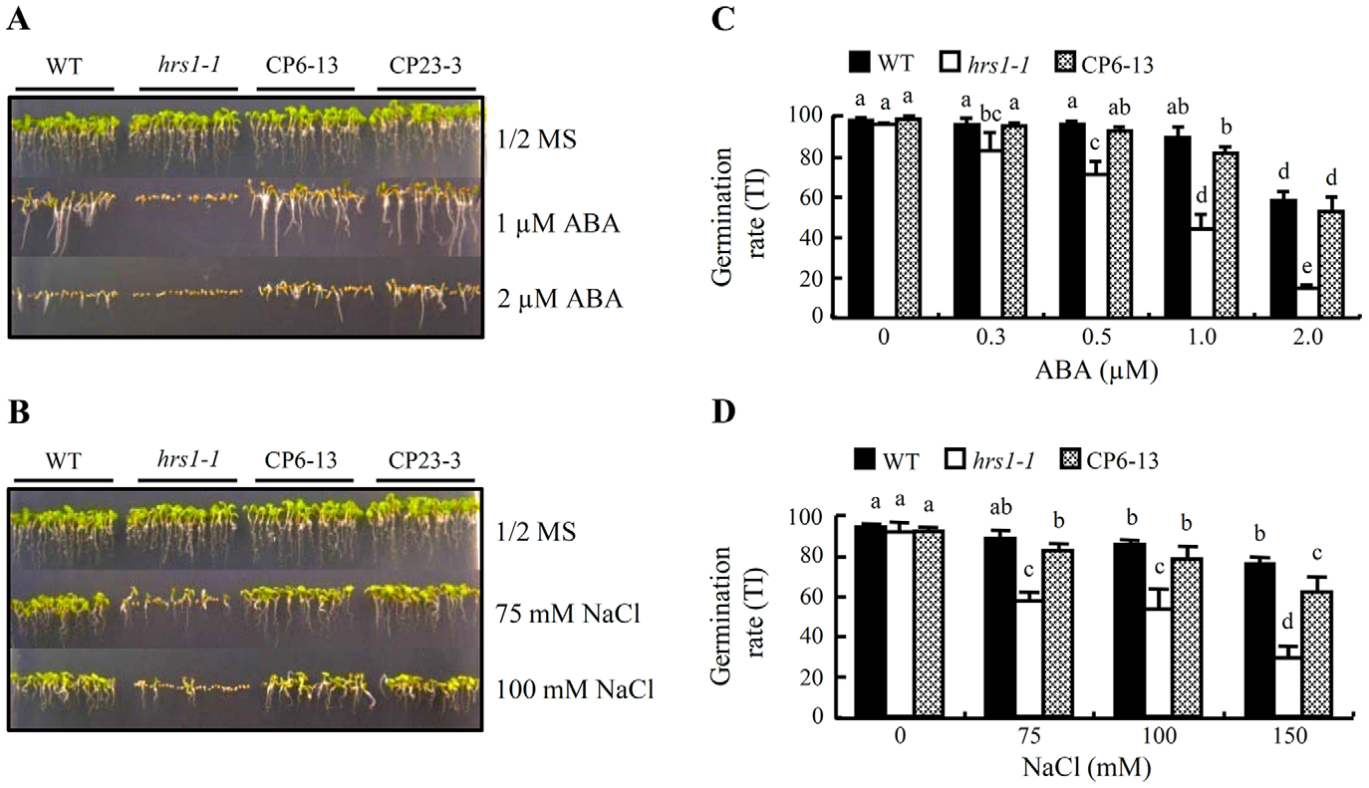
Effects of different concentrations of exogenous abscisic acid and NaCl on *hrs1-1* germination. (A, B) The more severe delay of *hrs1-1* germination, as compared to that of WT control and two CP lines (CP6-13 and CP23-3), conferred by increasing concentrations of abscisic acid (ABA) or NaCl added to the media. The data shown were recorded after 4 days of germination at 23°C, and are representative of five separate assays. (C, D) Comparisons of the germination rates among WT control, *hrs1-1* and CP line (CP6-13) on the media with the indicated concentrations of ABA or NaCl. Each germination rate value (TI, mean ± SD) was calculated using the measurements from triplicate samples after 6 days of germination at 23°C. The higher the value, the more rapid the germination proceeds. The data presented are all representative of three independent experiments, with the additional CP lines (CP19-1 and CP23-3) performing similarly as CP6-13 during the experiments. The means are labeled by different letters or letter combinations according to multiple statistical comparisons, and those labeled by one or more identical letters do not differ significantly from each other (*P*≤0.05).

The effects of different concentrations of ABA or NaCl on the germination behavior of different genotypes were further investigated. From Figure 2A and 2B, it is clear that the germination of *hrs1-1* was more strongly delayed by increasing concentrations of ABA or NaCl relative to that of WT control and two CP lines. To assess the effects of exogenous ABA and NaCl quantitatively, mean germination rate, represented by Timson index (TI) and reflecting the average velocity of germination [36], was calculated for the individual genotypes. The germination rates of WT control, *hrs1-1* and CP6-13 did not differ significantly from each other in the absence of ABA or NaCl, and no significant differences were found between the germination rates of WT control and CP6-13 on either ABA or NaCl containing media (Figure 2C and 2D). However, compared with WT control and CP6-13, *hrs1-1* was hypersensitive to the reduction of germination rate by rising concentrations of ABA (Figure 2C). Similarly, the germination rate of *hrs1-1* was more significantly lowered by increasing concentrations of NaCl (75 to 150 mM) than those of WT control and CP6-13 (Figure 2D).

Collectively, the data from the different sets of assays described above indicated that *hrs1-1* germination was transiently but significantly delayed compared to that of WT control and CP lines under normal conditions. The abnormality of *hrs1-1* germination could be severely exacerbated by ABA treatment or salt stress conferred by NaCl. Restoring *HRS1* function in the CP lines effectively corrected the defects of *hrs1-1* germination on either normal medium or those supplemented with ABA or NaCl.

### Molecular genetic analysis of *hrs1-1* germination

**Figure 3:**
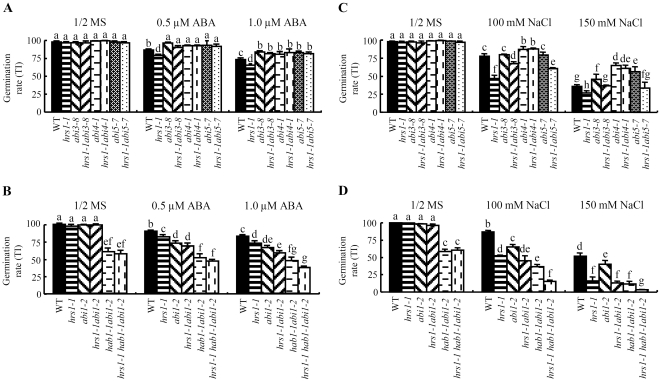
Genetic analysis of *hrs1-1* germination. Germination rate (TI, mean ± SD, each calculated using the measurements from triplicate samples) was used to compare the germination behavior of different genotypes. The datasets presented are each representative of at least three independent experiments. Based on multiple statistical comparisons, the means labeled by one or more identical letters do not differ significantly from each other (*P*≤0.05). (A) Comparisons of the germination rates of WT control, four single mutants (*hrs1-1*, *abi3-8*, *abi4-1* and *abi5-7*), and three double mutants (*hrs1-1abi3-8*, *hrs1-1abi4-1* and *hrs1-1abi5-7*) on 1/2 MS medium or those with two different concentrations of ABA. (B) Comparisons of the germination rates of WT control, two single mutants (*hrs1-1* and *abi1-2*), two double mutants (*hrs1-1abi1-2* and *hab1-1abi1-2*), and one triple mutant (*hrs1-1hab1-1abi1-2*) on 1/2 MS medium or those with two different concentrations of ABA. (C) The germination rates of WT control, four single mutants (*hrs1-1*, *abi3-8*, *abi4-1* and *abi5-7*), and three double mutants (*hrs1-1abi3-8*, *hrs1-1abi4-1* and *hrs1-1abi5-7*) on 1/2 MS medium or those containing two concentrations of NaCl. (D) The germination rates of WT control, two single mutants (*hrs1-1* and *abi1-2*), two double mutants (*hrs1-1abi1-2* and *hab1-1abi1-2*), and one triple mutant (*hrs1-1hab1-1abi1-2*) on 1/2 MS medium or those supplemented with two concentrations of NaCl.

Four sets of experiments were conducted in this investigation. First, the requirement of *ABI3*, *4* and *5* genes, which encode important positive regulators of ABA signaling pathway during seed germination [37]–[39], in the ABA hypersensitive germination of *hrs1-1* was examined. This was facilitated by the development of three double mutants lacking *HRS1* and *ABI3*, *ABI4* or *ABI5*. On 1/2 MS medium, the three double mutants (*hrs1-1abi3-8*, *hrs1-1abi4-1*, *hrs1-1abi5-7*) and *abi3-8*, *abi4-1* and *abi5-7* all germinated much faster than WT control, whereas *hrs1-1* germination again exhibited transient but significant delay (Figure S1). When germinated in the presence of ABA, *hrs1-1abi3-8*, *hrs1-1abi4-1*, and *hrs1-1abi5-7* did not exhibit enhanced ABA sensitivity as *hrs1-1* did (Figure 3A). Instead, their germination behavior resembled highly that of *abi3-8*, *abi4-1* and *abi5-7* in being relatively tolerant to exogenous ABA (Figure 3A).

Second, the genetic interactions between *HRS1* and the previously reported negative regulators of ABA signaling (such as *ABI1* and *HAB1*) were tested [15], [17], [18]. Two new mutants, e.g., *hrs1-1abi1-2* and *hrs1-1hab1-1abi1-2*, were developed, and their germination was compared to that of WT and the relevant parental lines (*hrs1-1*, *abi1-2* and *hab1-1abi1-2*). On 1/2 MS medium, only the germination rates of two genotypes (*hab1-1abi1-2*, *hrs1-1hab1-1abi1-2*) were significantly lower than that of WT control, but the two genotypes did not differ significantly from each other in germination behavior (Figure 3B). The presence of ABA in the medium decreased the germination of all six genotypes, but the germination of the five mutants was generally slower than that of WT control (Figure 3B). Consistent with earlier work [18], the germination rate of *hab1-1abi1-2* was significantly lower than that of *abi1-2* under ABA treatment (Figure 3B). Importantly, under both ABA concentrations, the germination rates of *hrs1-1*, *hrs1-1abi1-2* and *hrs1-1hab1-1abi1-2* differed significantly from each other, with those of *hrs1-1* and *hrs1-1hab1-1abi1-2* being the highest and lowest, respectively (Figure 3B). On the ABA containing media, the germination rate of *abi1-2* was generally lower than that of *hrs1-1*, and *abi1-2* and *hrs1-1abi1-2* did not differ substantially in germination (Figure 3B). Moreover, the germination rate of *hab1-1abi1-2* was lower than that of *hrs1-1abi1-2* (Figure 3B). The ABA concentrations for achieving 50% inhibition (IC50) of seed germination were estimated for WT control, *hrs1-1*, *abi1-2*, *hrs1-1abi1-2*, *hab1-1abi1-2* or *hrs1-1hab1-1abi1-2*, which were found to be 0.68, 0.42, 0.33, 0.25, 0.20 and 0.12 µM, respectively.

Third, the involvement of the positive and negative regulators of ABA signaling in *hrs1-1* germination behavior under NaCl stress was investigated using mainly the double and triple mutants described above. On the media containing 100 or 150 mM NaCl, the germination rates of the three double mutants lacking *HRS1* and *ABI3*, *4* or *5* were not decreased as significantly as that of *hrs1-1* (Figure 3C), whereas the germination rate of *hrs1-1hab1-1abi1-2* was significantly lower than those of WT control and the other four genotypes (*hrs1-1*, *abi1-2*, *hrs1-1abi1-2*, and *hab1-1abi1-2*) (Figure 3D). Moreover, on NaCl containing medium, the germination rate of *hrs1-1* was significantly lower than that of *abi1-2*, and the germination rate of *hrs1-1abi1-2* was more significantly decreased relative to that of *abi1-2* (Figure 3D). The germination rate of *hrs1-1abi1-2* was comparable to that of *hab1-1abi1-2* under NaCl treatment (Figure 3D).

**Figure 4:**
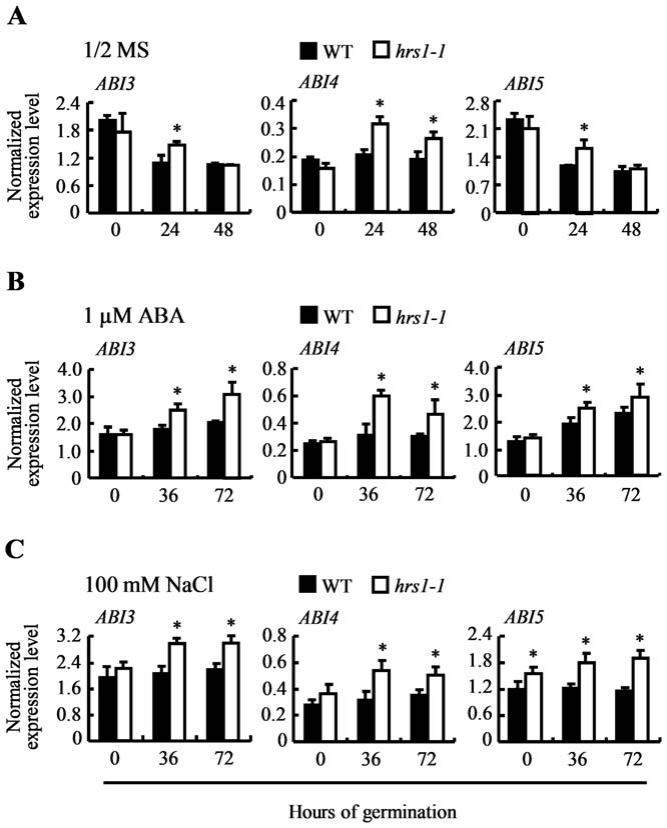
Analysis of *ABI3*, *ABI4* and *ABI5* expression during the germination of wild type control and *hrs1-1* seeds. Quantitative PCR was conducted with gene specific oligonucleotide primers. The amplification of *ACT8* (*At1g49240*, encoding *Arabidopsis* ACTIN8) served as the internal control. The normalized expression levels (means ± SD) were each calculated using the results from three technical repeats, and are representative of three independent experiments. (A–C) Comparisons of *ABI3*, *ABI4* and *ABI5* expression levels between wild type (WT) control and *hrs1-1* samples collected at the indicated time points during germination on 1/2 MS medium or the media supplemented with 1 µM ABA or 100 mM NaCl. Asterisk indicates statistical significance from WT control at *P*≤0.05.

Fourth, potential changes in the transcript levels of *ABI3*, *ABI4* and *ABI5* during the germination of WT control and *hrs1-1* under different conditions were compared using quantitative PCR. On 1/2 MS medium, the transcript levels of the three genes either decreased progressively (*ABI3*, *ABI5*) or did not show significant change (*ABI4*) from 0 to 48 HOG for WT control (Figure 4A). However, for *hrs1-1* germinating under the same condition, the decline in the transcript levels of *ABI3* and *ABI5* occurred more slowly (particularly at 24 HOG), and the transcript level of *ABI4* in *hrs1-1* was higher than that in WT control at 24 and 48 HOG (Figure 4A). On the medium with exogenous ABA (1 µM), the transcript levels of *ABI3* and *ABI5* did not show any decline for either WT control or *hrs1-1*, and were even substantially up-regulated at 36 and 72 HOG in the case of *hrs1-1* (Figure 4B). The transcript level of *ABI4*, although remained roughly stable in WT control, increased considerably for *hrs1-1* from 0 to 72 HOG on the medium containing 1 µM ABA (Figure 4B). The presence of 100 mM NaCl in the medium sustained the transcripts of *ABI3*, *ABI4* and *ABI5* at high levels from 0 to 72 HOG for either WT control or *hrs1-1*, but the transcript levels of the three genes were generally significantly higher in *hrs1-1* than in WT control (Figure 4C).

### Temporal and spatial patterns of *HRS1* expression during seed germination

**Figure 5:**
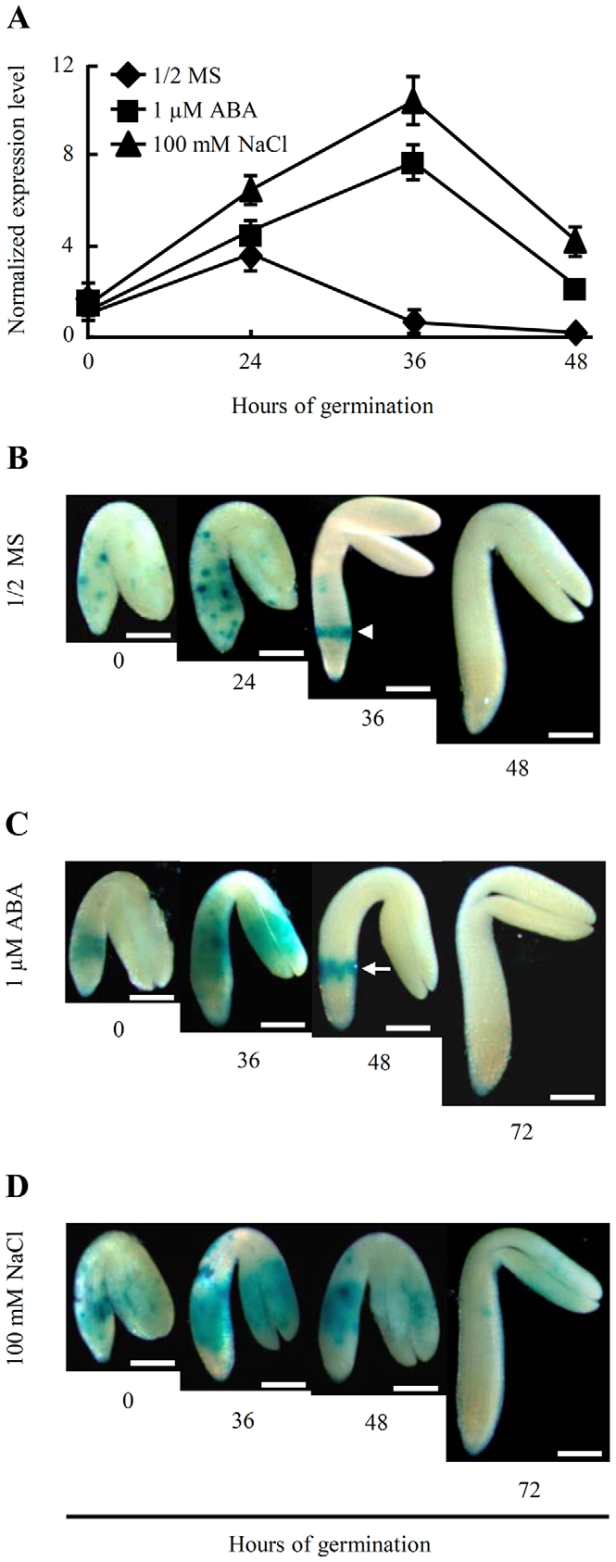
Temporal and spatial patterns of *HRS1* expression during seed germination. (A) Comparisons of *HRS1* expression levels at 0, 24, 36 and 48 HOG on 1/2 MS medium or those supplemented with 1 µM ABA or 100 mM NaCl by quantitative PCR. The amplification of *ACT8* (*At1g49240*) served as the internal control. The normalized expression levels (means ± SD) were each determined using the data from three technical repeats, and are typical of four independent experiments. (B–D) The spatial patterns of *HRS1* expression during *Arabidopsis* seed germination on 1/2 MS medium or those supplemented with 1 µM ABA or 100 mM NaCl investigated using the *HRS1* promoter::GUS reporter line RL3-11. *HRS1* expression, detected at the designated time points, is indicated by the blue signals generated by histochemical staining of GUS activity. The arrowhead and arrow indicate the zone of strong *HRS1* expression detected at 36 HOG on 1/2 MS medium or 48 HOG on ABA containing medium. The three sets of data shown are each representative of four independent GUS staining experiments. Similar results were obtained with the two additional reporter lines RL4-9 and RL5-1. Bars = 0.4 mm.

The after-ripened seeds of WT control and three independent promoter:: β-glucuronidase (GUS) reporter lines of *HRS1* were cold stratified as described above, followed by transfer to 23°C to allow germination under different conditions. *HRS1* expression patterns were investigated using quantitative PCR (for WT seeds) or histochemical staining of GUS activity (for the seeds of promoter::GUS reporter lines). From the quantitative PCR data shown in Figure 5A, it is evident that, on 1/2 MS medium, *HRS1* expression was relatively high at 24 HOG, but declined thereafter and became undetectable at 48 HOG. On the media supplemented with 1 µM ABA or 100 mM NaCl, *HRS1* expression increased from 0 to 36 HOG, but decreased rapidly from 36 to 48 HOG (Figure 5A). Compared to germination on 1/2 MS medium, the presence of exogenous ABA or NaCl in the medium significantly up-regulated *HRS1* transcript level at 24, 36 and 48 HOG, with the scale of the up-regulation being much larger by NaCl (Figure 5A). Furthermore, the timing of the decline in *HRS1* expression was delayed by exogenous ABA or NaCl relative to that observed on 1/2 MS medium (Figure 5A).

The typical *HRS1* expression patterns, revealed by histochemical staining of GUS activity and indicated by blue signals, are shown in Figure 5B to 5D. On 1/2 MS medium, *HRS1* was expressed mainly in the hypocotyl and root precursor cells of embryo axis at 0, 24 and 36 HOG, with the highest expression level detected around 24 HOG (Figure 5B). No *HRS1* expression was found in the germinated embryos collected at 48 HOG (Figure 5B). A low level of *HRS1* expression also occurred in the cotyledonary cells at 0 and 24 HOG, which was undetectable at 36 and 48 HOG (Figure 5B). *HRS1* expression in embryo axis at 0 and 24 HOG was characterized by a punctuate pattern, which turned largely confined to a discrete zone by 36 HOG (Figure 5B, indicated by arrowhead). The main location of *HRS1* expression was found also in embryo axis during germination on the media containing ABA or NaCl, but *HRS1* was more highly and more broadly expressed in embryo axis under these two conditions (Figure 5C and 5D). A distinct zone of strong *HRS1* expression was also observed in embryo axis in the presence of ABA (Figure 5C, marked by arrow), but it was not detected until after 40 HOG. A clear zone of strong *HRS1* expression in embryo axis was not found in the seeds germinating on NaCl containing medium at 48 HOG, instead, a substantially more expanded *HRS1* expression area was observed in embryo axis at this time point (Figure 5D). At 72 HOG, *HRS1* expression was undetectable, or at a very low level, in the germinated embryos on either ABA or NaCl supplemented media (Figure 5C and 5D). The three promoter::GUS reporter lines gave highly similar results.

### Examination of cell elongation growth in germinating embryo axis

**Figure 6:**
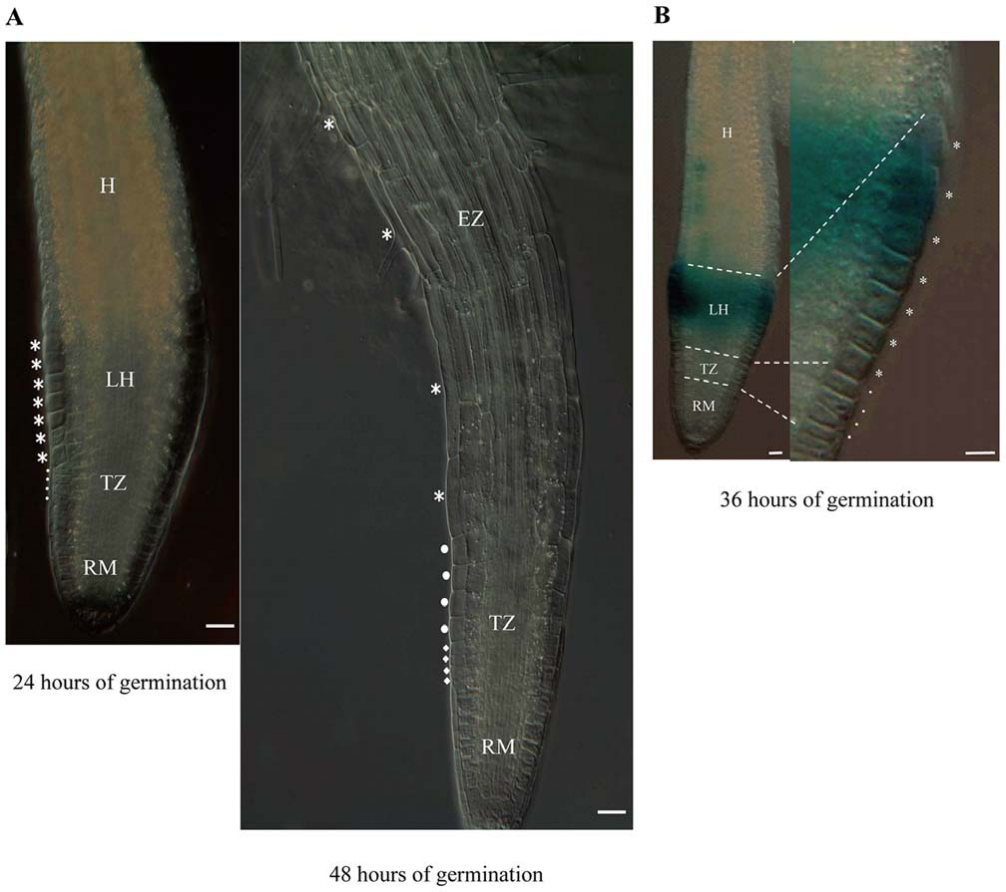
Elongation growth of the cells in lower hypocotyl and transition zone, and the occurrence of strong *HRS1* expression in lower hypocotyl cells, during *Arabidopsis* seed germination. Lower hypocotyl (LH) cells are marked by asterisks, whereas the cells in transition zone (TZ) are labeled by filled dots. (A) Comparison of LH and TZ cells in the radicle samples collected at 24 or 48 HOG. Both LH and TZ cells were elongated from 24 to 48 HOG, with the magnitude of the elongation being substantially larger for LH cells. At 48 HOG (immediately post germination), an elongation zone (EZ), differentiated mainly from elongated LH cells, was found in the young seedling root. In addition, four newly developed TZ cells (indicated by diamonds) were observed on top of the root meristematic region. (B) The occurrence of strong HRS1 expression in the LH cells (of the promoter::GUS reporter line RL3-11) as indicated by the blue signals produced by histochemical staining of GUS activity at 36 HOG. The expression pattern shown was typical of four separate staining experiments, and was found for all three independent promoter::GUS reporter lines of *HRS1*. EZ, elongation zone; H, hypocotyl; LH, lower hypocotyl; RM, root meristem; TZ, transition zone. Bars = 50 μm.

In WT seeds germinating on 1/2 MS medium (i.e., prior to radicle emergence), four morphologically distinguishable regions, e.g., hypocotyl (H), lower hypocotyl (LH), transition zone (TZ) and root meristem (RM), could be distinguished in the radicle half of embryo axis (Figure 6A, left panel), with the cells in LH being generally much larger than those in TZ (Figure 6A, left panel, LH and TZ cells were labeled by asterisk and dot, respectively). Consistent with earlier work [26], we found that, during the course of germination, the cells located in LH and TZ underwent substantial elongation (Figure 6A). By 48 HOG (i.e., after radicle emergence), an elongation zone (EZ, Figure 6A, right panel), differentiated mainly from elongated LH cells, was formed in the young seedling root. Interestingly, the discrete zone of strong *HRS1* expression in embryo axis, which was observed around 36 HOG on 1/2 MS medium (Figure 5B), corresponded to LH (Figure 6B).

**Figure 7:**
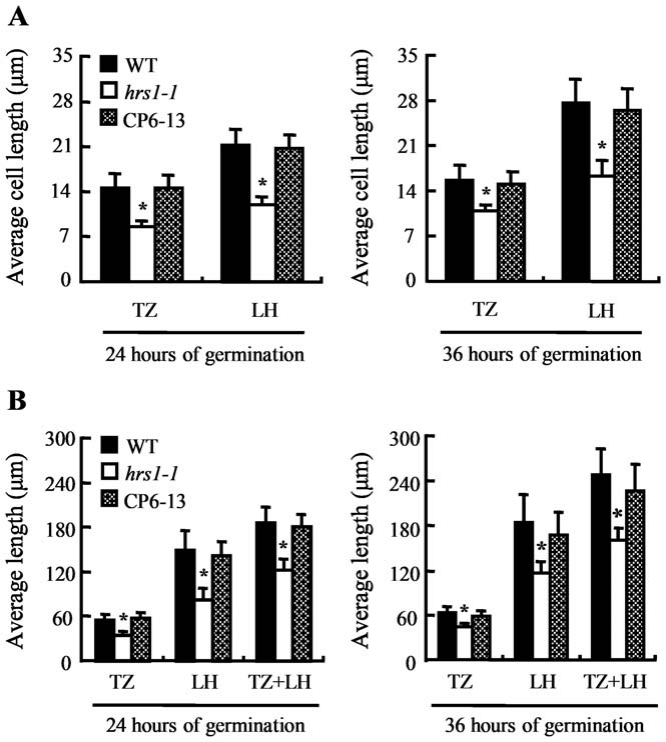
Examination of cell elongation growth in germinating embryo axis. The seeds of WT control, *hrs1-1* and CP line (CP6-13) were cold stratified for 48 h, and then allowed to germinate on 1/2 MS medium at 23°C. The measurements were made at 24 and 36 HOG, respectively. (A) The average cell length values (means ± SD, *n* = 30∼35 samples measured per data point) in transition zone (TZ) and lower hypocotyl (LH) in WT control, *hrs1-1* and CP6-13. The data displayed are representative of three separate experiments. (B) The average length values (means ± SD, *n* = 33∼37 samples measured per data point) of TZ, LH, and TZ plus LH in WT control, *hrs1-1* and CP6-13. The data shown are typical of three independent experiments. The additional CP lines (CP19-1 and CP23-3) behaved similarly as CP6-13 during these experiments. Asterisk indicates statistical significance from WT control and CP line at *P*≤0.05.

Based on the above observations, we focused on LH and TZ cells for comparing the elongation growth in germinating embryo axis among WT control, *hrs1-1* and CP6-13 seeds. The embryo axis samples collected at 24 or 36 HOG on 1/2 MS medium were used as representatives for this analysis, and the longitudinal length of the cells in LH and TZ was individually measured for each embryo axis sample. As shown in Figure 7A, the average length of LH and TZ cells was all significantly longer in WT control and CP6-13 than in *hrs1-1* at either 24 or 36 HOG. In line with this result, the average size of both LH and TZ as well as their combined size were all significantly larger in WT control and CP6-13 than in *hrs1-1* (Figure 7B). The effects of exogenous ABA or NaCl on the elongation of LH and TZ cells in *hrs1-1* were not assessed in this work, because the germination of *hrs1-1* seeds in the presence of ABA or NaCl occurred highly asynchronously, making it difficult to accurately measure the length of LH and TZ cells.

**Figure 8:**
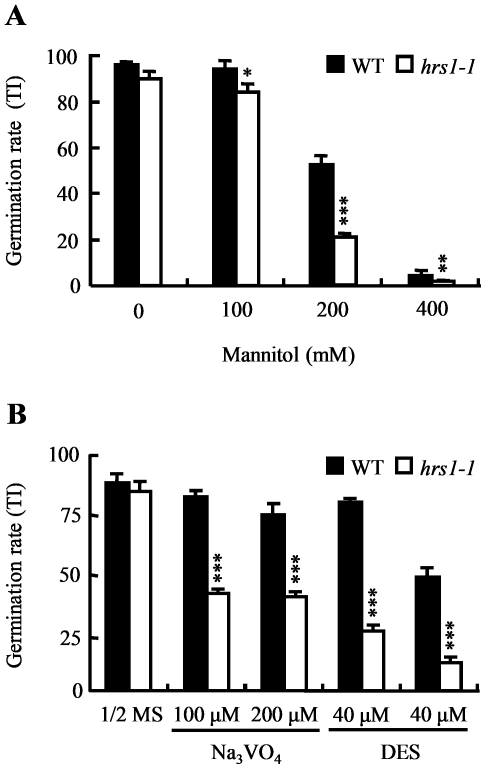
The effects of inhibitor treatment on the germination of wild type control and *hrs1-1*. The seeds of wild type (WT) control and *hrs1-1* were cold stratified for 48 h, followed by germination on the media without, or with the indicated concentrations of, mannitol, Na_3_VO_4_ or DES. The germination rate (TI, mean ± SD, each calculated with the results of triplicate samples) was recorded after six days of germination at 23°C. The data sets displayed are each typical of four independent experiments. (A) The germination rates of WT control and *hrs1-1* on the media without (0), or with three concentrations (100, 200 or 400 mM) of, mannitol. (B) The germination rates of WT control and *hrs1-1* on 1/2 MS medium or the media with two different concentrations of Na_3_VO_4_ or DES. Single, double and triple asterisks indicate statistical significance from WT control at *P*≤0.05, 0.01, and 0.001, respectively.

The above data led us to examine if *hrs1-1* germination might be more severely affected than that of WT control by the compounds that are known to inhibit cell elongation during seed germination. Previous studies have shown that mannitol is a potent inhibitor of cell elongation growth in germinating plant seeds [40]. Orthovanadate (Na_3_VO_4_) and diethylstilbestrol (DES) have been found to inhibit cell elongation during seed germination by inhibiting plasmalemma H^+^-ATPase in both monocotyledonous and dicotyledonous plants [41], [42]. As shown in Figure 8A, the germination rate of *hrs1-1* was more significantly reduced by the presence of mannitol in 1/2 MS medium than that of WT control, especially at higher mannitol concentrations (≥200 mM). The germination rate of *hrs1-1* was also more significantly decreased than that of WT control by either Na_3_VO_4_ or DES (Figure 8B).

### Analysis of plasmalemma H^+^-ATPase activity during seed germination

**Figure 9:**
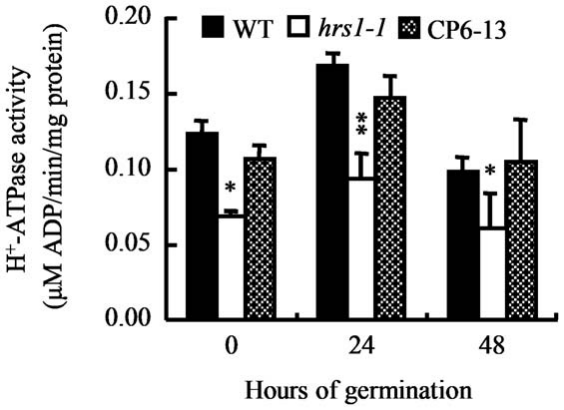
Analysis of plasmalemma H^+^-ATPase activity during the germination of wild type control, *hrs1-1* and complementation line. The seeds of wild type (WT) control, *hrs1-1* and complementation line CP6-13 were cold stratified for 48 h, followed by germination on 1/2 MS medium at 23°C. The levels of H^+^-ATPase activity (means ± SD, each calculated with the results of triplicate samples) were determined using plasma membrane enrich fractions prepared from the seed samples taken at the indicated time points. The data shown are typical of three independent experiments. The results obtained with the additional CP lines (CP19-1 and CP23-3) were highly similar to those determined for CP6-13 during the experiments. Single and double asterisks indicate statistical significance from WT control at *P*≤0.05 and 0.01, respectively.

**Figure 10:**
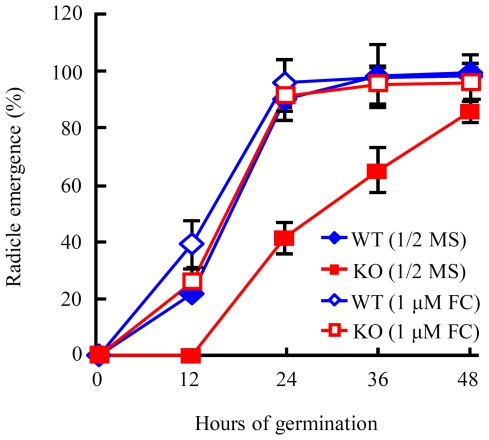
The effects of fusicoccin treatment on the germination of wild type control and *hrs1-1*. The seeds of wild type (WT) control and *hrs1-1* (KO) were cold stratified for 48 h, followed by germination on 1/2 MS medium or that supplemented with 1 µM fusicoccin (FC). The percentages of radicle emergence (means ± SD) at the indicated time points were each calculated using the results from triplicate samples. The data shown are typical of three separate germination experiments.

The data displayed in Figure 8B led us to examine if there might be difference in the plasmalemma H^+^-ATPase activity level in the germinating seeds of WT control and *hrs1-1*. The seeds of WT control, *hrs1-1* and CP6-13 were cold stratified and germinated on 1/2 MS medium as described above. Plasmalemma H^+^-ATPase activity levels were assayed using plasma membrane enriched samples prepared from the seeds collected at representative time points of the germination course. The levels of plasmalemma H^+^-ATPase activity in *hrs1-1* samples were generally and significantly lower than those exhibited by WT control or CP6-13 samples (Figure 9). However, WT and CP samples did not differ significantly in their levels of plasmalemma H^+^-ATPase activity (Figure 9). The data depicted in Figure 8B and Figure 9 propelled us to test the effect of fusicoccin (FC) on the germination of *hrs1-1*. FC has been demonstrated to activate plasmalemma H^+^-ATPase in a variety of plant tissues [43], [44]. As displayed in Figure 10, the addition of FC to 1/2 MS medium did not significantly influence the germination of WT control, but almost completely corrected the transient delay in *hrs1-1* germination.

## Discussion

In this work, we studied the function of *HRS1* during *Arabidopsis* seed germination by combining molecular genetic, cell biological and physiological approaches. From the transient and prolonged delays exhibited by *hrs1-1* germination on 1/2 MS medium or those supplemented with different concentrations of NaCl, and the correction of the germination defects in *hrs1-1* CP lines, we conclude that *HRS1* is needed for efficient seed germination in *Arabidopsis* under either normal or salt stress conditions, which extends substantially the brief observation made previously on the transient seed germination delay after knocking out *HRS1* function [34].

### *HRS1* is a novel negative regulator of abscisic acid signaling in germinating embryo axis

A combined consideration of the data obtained by germinating relevant *Arabidopsis* lines on 1/2 MS medium or that supplemented with ABA generates several lines of evidence for *HRS1* functioning as a negative regulator of ABA signaling in germinating embryo axis. First, *hrs1-1* germination was hypersensitive to exogenous ABA. Furthermore, this hypersensitivity required the key positive regulators of ABA signaling (i.e., *ABI3*, *4* and *5*), and was accompanied by the relatively higher transcript levels of *ABI3*, *4* and *5* in *hrs1-1* germinating seeds as compared to those in WT control. Together, these data indicate that the loss of *HRS1* function leads to enhanced ABA signaling during seed germination. Second, simultaneous inactivation of *HRS1* and the well established negative regulators of ABA signaling *ABI1* and *HAB1* caused further decreases in seed germination rate. The magnitude of the germination rate reduction was generally highest in the triple (*hrs1-1hab1-1abi1-2*), intermediate in the double (*hrs1-1abi1-2*, *hab1-1abi1-2*), and relatively low in the single mutants (*hrs1-1*, *abi1-2*), which is in line with the increasing IC50 values of ABA for the three categories of mutants. Moreover, like *ABI1* and *HAB1*, *HRS1* expression level in germinating seeds was significantly up-regulated by exogenous ABA. Collectively, these data indicate functional similarity and additive interactions among *HRS1*, *ABI1* and *HAB1* in regulating ABA signaling during seed germination. Third, *HRS1* was predominantly expressed in embryo axis during seed germination. This was indicated by the *HRS1* expression detected in the hypocotyl and root precursor cells, as well as the period of relatively strong *HRS1* expression occurred in LH cells, during the main course of seed germination. Therefore, the main site of *HRS1* function during seed germination is embryo axis. We did not observe a period of intense *HRS1* expression in TZ cells (as that found for LH cells) in this work, which might be due to the highly dynamic and transient nature of *HRS1* expression in the different regions of germinating embryo axis.

On the basis that *HRS1* acts as a negative regulator of ABA signaling in germinating embryo axis, it is possible that, in *hrs1-1* seeds germinating on 1/2 MS medium, there may exist abnormal up-regulation of ABA signaling in embryo axis because of the lack of the negative regulation conferred by *HRS1*. However, owing to the overall trend of decline in ABA signaling and function during seed germination under normal conditions [4], [8], the upsurge of ABA signaling in *hrs1-1* embryo axis germinating on 1/2 MS medium is likely to be temporary, thus delaying *hrs1-1* germination only transiently. But in the presence of exogenous and physiological concentrations of ABA, which tends to stimulate ABA signaling and function in plant cells, the deficiency of the negative regulation by *HRS1* may lead to a more drastic elevation of ABA signaling in *hrs1-1* germinating embryo axis (as compared to that occurred on 1/2 MS medium), thus causing more prolonged delay of *hrs1-1* germination and more severe reduction in *hrs1-1* germination rate.

From the discussion above, it is reasonable to suggest that *HRS1* is necessary for maintaining a normal level of ABA signaling in germinating embryo axis. Furthermore, *HRS1* may act upstream of *ABI3*, *4* and *5*, because the enhanced ABA signaling in *hrs1-1* germinating embryo axis required the three genes. Although we detected certain functional similarity and additive interactions among *HRS1*, *ABI1* and *HAB1* in negatively regulating ABA signaling during seed germination, further work is needed to investigate if the three genes may act through overlapping or independent pathways. Nevertheless, *HRS1* may not be as potent as *ABI1* in the negative regulation of ABA signaling during seed germination on ABA containing medium, because 1) the decrease of germination rate exhibited by *abi1-2* was significantly larger than that by *hrs1-1*, 2) the mutation of *HRS1* in *abi1-2* background did not further decrease germination rate as compared to that of *abi1-2*, and 3) the IC50 value of ABA was substantially higher for *hrs1-1* than for *abi1-2*. Since the germination rate of *hab1-1abi1-2* was significantly lower than that of *hrs1-1abi1-2* on either normal or ABA containing media, it is possible that *HAB1* may also be a stronger negative regulator of ABA signaling than *HRS1* during *Arabidopsis* seed germination. However, this possibility needs to be verified by more detailed comparisons of the germination behavior between *hrs1-1* and *hab1-1* in future research. Because of its dynamic expression and function in germinating embryo axis (revealed in this work) and potential transcription repression activity demonstrated previously [35], *HRS1* is unique from the formerly characterized negative regulators of ABA signaling that encode type 2C protein phosphatases (such as ABI1 and HAB1). Together, the features described above also make *HRS1* distinct from *RACK1*, *AZF2* and *MFT*, which have recently been found to participate in the negative regulation of ABA signaling during *Arabidopsis* seed germination [30]–[32], but encode protein products that are very different from that specified by *HRS1*.

### *HRS1* may facilitate proper elongation of the cells located in lower hypocotyl and transition zone and normal function of plasmalemma H^+^-ATPase during seed germination

In agreement with earlier work [26], we found that the cells located in LH and TZ were the ones undergoing elongation and contributed to the growth of embryo axis (as indicated by increases in the size of LH and TZ) and radicle emergence during the germination of WT seeds. But, more importantly, we uncovered for the first time that the loss of *HRS1* function impaired not only the elongation of LH and TZ cells but also the growth of embryo axis in *hrs1-1* seeds germinating on 1/2 MS medium. We further showed that the level of plasmalemma H^+^-ATPase activity was significantly reduced in *hrs1-1* germinating seeds (as compared to that in WT controls). Because the defective elongation of LH and TZ cells, the decrease of plasmalemma H^+^-ATPase activity, and the transient delay exhibited by *hrs1-1* seed germination were all corrected in CP lines, it is highly likely that the facilitation of LH and TZ cell elongation and plasmalemma H^+^-ATPase function may be important for the regulation of seed germination by *HRS1*. This proposition is consistent with the finding that *HRS1* was predominantly expressed in embryo axis cells (including those in LH and TZ regions) during the main course of seed germination. It is also supported by the following lines of physiological evidence generated in this work. First, the treatment with a cell elongation inhibitor (mannitol) decreased the germination rate of *hrs1-1* more steeply than that of WT control. Second, the germination rate of *hrs1-1* was more strongly lowered than that of WT control by the application of the inhibitors of plasmalemma H^+^-ATPase (Na_3_VO_4_ or DES). Finally, the supply of an activator of plasmalemma H^+^-ATPase (FC) almost completely mitigated the transient delay of *hrs1-1* germination on 1/2 MS medium.

How may *HRS1* exert its influence on cell elongation and plasmalemma H^+^-ATPase activity in germinating embryo axis? Based on the data collected here and those published previously [8], [27], [44], it is probable that, during the germination of *Arabidopsis* seeds under normal conditions, a gradual decline of ABA content and signaling in germinating embryo axis is essential for the proper function of plasmalemma H^+^-ATPase activity, which leads to apoplastic acidification, efficient elongation of LH and TZ cells, and timely germination completion. As a negative regulator of ABA signaling in germinating embryo axis, the action of *HRS1* contributes to the decrease of ABA function, and thus facilitates the proper function of plasmalemma H^+^-ATPase, the efficient elongation of LH and TZ cells, and the timely progression of germination. However, in the absence of *HRS1*, an abnormal increase of ABA signaling in germinating embryo axis may interfere with plasmalemma H^+^-ATPase function and subsequent cell elongation growth. To further investigate this possibility in the future, it is interesting to note that ABA has been shown to inhibit blue-light induced plasma membrane H^+^-ATPase activity in the guard cells by modulating H^+^-ATPase phosphorylation status [45], [46]. Therefore, it will be important to examine if a similar mechanism might be involved in the impairment of plasmalemma H^+^-ATPase activity during *hrs1-1* germination. Alternatively, the abnormal uplift of ABA signaling in germinating embryo axis caused by functional deficiency of *HRS1* might lead to enhanced auxin signaling, thus disrupting plasma membrane H^+^-ATPase activity and elongation growth in embryo axis cells. Enhanced auxin signaling has been found responsible for ABA mediated inhibition of cell elongation in the young seedling root immediately post germination [22]. Moreover, there is also molecular evidence that abnormal enhancement of auxin signaling inhibits *Arabidopsis* seed germination [47]. Consequently, it will also be worthwhile to investigate if auxin signaling may be abnormally up-regulated in *hrs1-1* germinating embryo axis, and its potential involvement in the control of plasmalemma H^+^-ATPase activity and the elongation of LH and TZ cells during seed germination in future research.

Among the previously characterized negative regulators that affect ABA signaling during *Arabidopsis* seed germination, *AZF2* and *MFT* have been shown to be expressed in germinating embryo axis [31], [32], with *MFT* expression further found in TZ cells prior to radicle emergence [32]. In the light of this work, it becomes pertinent to investigate if the function of *AZF2* and *MFT* during seed germination may also be due to the facilitation of normal function of plasmalemma H^+^-ATPase and efficient LH and TZ cell elongation in germinating embryo axis. It will also be interesting to study if the mechanisms behind the regulation of seed germination by *ABI1* and related *PP2C* genes may bear certain similarity to those adopted by *HRS1* (i.e., negative regulation of ABA signaling and facilitation of plasmalemma H^+^-ATPase function and cell elongation growth in germinating embryo axis).

Although the data collected in this work support a role of *HRS1* in the regulation of radicle growth by facilitating plasmalemma H^+^-ATPase function and LH and TZ cell elongation in germinating embryo axis, the potential involvement of *HRS1* in other processes vital for timely seed germination remains to be investigated. For example, it has been well demonstrated that endosperm weakening and rupture constitute an essential step in seed germination, and that ABA plays a significant regulatory role in endosperm weakening and rupture [24], [25]. In addition to plasmalemma H^+^-ATPase, recent studies indicate that production of reactive oxygen species (ROS) also has a positive role for seed germination by promoting endosperm rupture and radicle growth [48], [49]. However, ROS production in germinating seeds is inhibited by ABA, and this inhibition accompanies the delay of endosperm weakening and rupture by ABA treatment [49]. In view of these important findings and the negative regulation of ABA signaling by *HRS1* uncovered in this work, it will be interesting to analyze if *HRS1* may also be involved in the control of ROS accumulation and endosperm weakening and rupture in germinating seeds in the future.

### *HRS1* may be involved in the negative regulation of abscisic acid signaling during seed germination under salt stress conditions

Three lines of evidence suggest that *HRS1* may also be involved in the negative regulation of ABA signaling in embryo axis during seed germination on NaCl containing medium. First, the decrease in *hrs1-1* germination rate by NaCl depended upon the existence of *ABI3*, *4* and *5* genes, and was associated with the sustained transcript levels of the three genes. Second, *hrs1-1* germination defect in the presence of NaCl was further aggravated by combining together the mutations of *HRS1*, *ABI1* and *HAB1*. Third, NaCl significantly increased the expression level of *HRS1*, and *HRS1* maintained its expression predominantly in embryo axis during the main course of germination.

Interestingly, *hrs1-1* germination was more severely affected on NaCl containing medium relative to that on 1/2 MS medium or the medium with exogenous ABA. This phenomenon may be due to at least two possibilities. First, the negative regulation of ABA signaling imparted by *HRS1* may become even more important under salt conditions. This suggestion is consistent with the more significantly increased *HRS1* transcript level and the more severe delay in the decline of *HRS1* expression in germinating embryo axis in the presence of NaCl. It is also in line with the observations that the extent of germination rate reduction exhibited by *hrs1-1* was significantly larger than that of *abi1-2*, and that the mutation of *HRS1* in *abi1-2* background caused further decrease in germination rate relative to that of *abi1-2*, on the media supplemented with 100 or 150 mM NaCl. Second, the more severe decrease in *hrs1-1* germination on NaCl containing medium might be augmented by complex changes, involving not only alteration of ABA signaling in embryo axis but also increased vulnerability to ion imbalance and water deficit injuries brought about by salt stress [50]. Further experimentation is required to investigate if *HRS1* might be involved in regulating both ABA signaling and cell responses to salt injuries during seed germination.

In either of the two possible scenarios above, the severe decrease of *hrs1-1* germination on NaCl containing medium may be partially connected with significant enhancement of ABA signaling in germinating embryo axis, which bears similarity to the mechanism controlling the temporary arrest of germinated *Arabidopsis* embryos under adverse conditions [21]. Therefore, in *Arabidopsis*, the germination and post germination growth may all be sensitive to inhibition by abiotic stress through, at least partly, enhanced ABA signaling in embryo axis. Post germination arrest of *Arabidopsis* embryos under adverse conditions by increased ABA signaling is an important adaption of plants to abiotic stresses [21]. A similar adaptive strategy may also operate during germination. However, its effectiveness requires the normal function of the negative regulators of ABA signaling (such as *HRS1*) in embryo axis.

In summary, we have shown that *HRS1* is a novel negative regulator of ABA signaling during *Arabidopsis* seed germination. It may participate in the suppression of ABA signaling in germinating embryo axis, which in turn promotes the germination of *Arabidopsis* seeds in either normal or salt stress environments. Our cell biological and physiological experiments suggest that *HRS1* may facilitate the proper function of plasmalemma H^+^-ATPase and the efficient elongation of LH and TZ cells in germinating embryo axis during its regulation of seed germination under normal conditions. Further work is required to achieve a more comprehensive understanding of the potential involvement of *HRS1* in the different molecular and physiological processes during *Arabidopsis* seed germination. More detailed studies are also needed to unveil the relationships among *HRS1* and other components of ABA signaling pathway and the targets of *HRS1* transcriptional repression activity. The findings made in this work, plus the existence of *HRS1* homologs in both monocotyledonous and dicotyledonous plants (Figure S2), suggest that *HRS1* may continue to aid deeper investigations into the complex genetic, cell biological and physiological mechanisms controlling embryo axis growth and timely seed germination in higher plants in the future.

## Materials and Methods

### Plant materials

All *Arabidopsis thaliana* materials used in this work were in the Col-0 ecotype background. The oligonucleotide primers used in this work are listed in Table S1. The knockout mutant *hrs1-1* and the three independent promoter::GUS reporter lines of *HRS1* were reported previously [33]. The complementation lines were prepared by transgenic expression of WT *HRS1* coding sequence under *HRS1* native promoter in *hrs1-1*. Briefly, the 5′ flanking sequence of *HRS1* (1617 bp), amplified by PCR from Col-0 genomic DNA sample using the primers pHRS1-F and pHRS1-R (Table S1), was inserted between the *Sac*I and *Hind*III sites of the pJIT163 vector (http://www.pgreen.ac.uk/), giving rise to pHRS1. The 1615 bp genomic open reading frame of *HRS1* was amplified with the primers HRS1-F1 and HRS1-R1, and was inserted into the *Hind*III and *Bam*HI sites of pHRS1, producing pHRS1::HRS1. The HRS1::HRS1 expression cassette (with the *nopaline synthase* transcription termination sequence derived from pJIT163) was transferred into pCAMBIA1300, resulting in the T-DNA construct p1300HRS1::HRS1. This construct was used to transform *hrs1-1* using the floral dip method [51], with homozygous transgenic lines identified in the T3 generation. Three independent complementation lines (named as CP6-13, CP19-1 and CP23-3 respectively) were developed. They gave highly similar results in the experiments described in this work.

Double and triple mutants lacking *HRS1* and one or more of the genes encoding the positive (*ABI3*, *4* and *5*) or negative (*ABI1* and *HAB1*) regulators of ABA signaling were developed. Homozygous *abi1-2*, *hab1-1*, and *hab1-1abi1-2* lines were obtained from Dr. Pedro Rodriguez [18]. The *abi1-2* allele was a loss-of-function mutant caused by T-DNA insertion, and differed from the gain-of-function allele *abi1-1* in being sensitive to exogenous ABA [18]. The homozygous seeds of *abi3-8* and *abi5-7* were obtained from Dr. Eiji Nambara [52]. The *abi4-1* seeds were obtained from *Arabidopsis* Biological Resource Center (http://abrc.osu.edu/). To generate *hrs1-1abi1-2*, *hrs1-1hab1-1abi1-2*, *hrs1-1abi3-8*, *hrs1-1abi4-1*, *hrs1-1abi5-7* mutants, the pollen grains of *abi1-2*, *hab1-1abi1-2*, *abi3-8*, *abi4-1* or *abi5-7* plants were transferred to the stigmas of the emasculated flowers of homozygous *hrs1-1*. The F1 plants were allowed to self-pollinate. The F2 plants were genotyped by PCR using either dominant (for *hrs1-1abi1-2* and *hrs1-1hab1-1abi1-2*) or co-dominant dCAPS markers (for *hrs1-1abi3-8, hrs1-1abi4-1* and *hrs1-1abi5-7*). The F3 seeds with the desired genotype underwent another cycle of multiplication, yielding sufficient F4 seeds for the subsequent experiments. The primers used for mutant identification are listed in Table S1.

### Germination assay

The seeds of WT control and the different transgenic or mutant lines, harvested at the same time and stored at 25°C for at least four weeks, were used for germination assays. According to the purpose of the experiments, germination assays were conducted on either 1/2 MS medium, or those supplemented with the designated chemical compound (i.e., ABA, NaCl, mannitol, Na_3_VO_4_, diethylstilbestrol, or fusicoccin). 1/2 MS medium was composed of half strength Murashige and Skoog (MS) basal salts (including vitamins, purchased from Duchefa Biochemie BV, Netherlands) [53], 0.1% 2-(N-morpholino)ethanesulfonic acid, 1% sucrose and 1% agar. The pH was adjusted to 5.8 with potassium hydroxide before autoclaving. Seeds were surface sterilized, plated on the desired medium and stratified at 4°C for 2 days, followed by transfer to a growth chamber (23°C, 16 h light/8 h dark photoperiod) to allow germination to start. Each germination assay was conducted in triplicates, with about 50 seeds used per replicate per genotype. Seeds were judged as germinated when testa and endosperm rupture and radicle protrusion were observed [24], [25]. For comparing the time courses of germination of different genotypes on 1/2 MS medium or those supplemented with ABA, NaCl or fusicoccin, the percentages of radicle emergence were determined at the selected time points. In other assays, germination rate, measured as Timson's index (TI) and reflecting germination velocity of seeds [36], was determined. For calculating TI, the formula TI = Σ (G/t) was used, where G is the germination percentage (scored daily for 6 days), and t is the total days of germination. The value of TI may vary from 100 (100% of seeds germinated in the first day) to 0 (no seeds germinated during the 6 days assay period). The higher is the TI value the more rapid is the germination. The ABA concentrations required to obtain 50% inhibition of seed germination of WT control, *hrs1-1*, *abi1-2*, *hrs1-1abi1-2*, *hab1-1abi1-2* or *hrs1-1hab1-1abi1-2* were estimated as detailed previously [18].

### Quantitative polymerase chain reaction assay

WT and *hrs1-1* seeds were sown on 1/2 MS plates or those supplemented with 1 µM ABA or 100 mM NaCl, followed by cold stratification and germination as described above. Samples were collected at the selected time points. Total RNA extraction, cDNA synthesis and quantitative PCR assays were carried out as described in an earlier work [54]. The normalized expression levels of the target genes were estimated as described previously [55]. At least three independent biological replicates were performed in order to check the reproducibility of the data. The gene specific primers used for quantitative PCR were listed in Table S1.

### Histochemical staining of β-glucuronidase activity and microscopy

The seeds of three independent *HRS1* promoter::GUS reporter lines (designated as RL3-11, RL4-9 and RL5-1) were germinated on 1/2 MS plates or those supplemented with 1 µM ABA or 100 mM NaCl as described above. Sample materials were collected at 0, 24, 36 and 48 HOG, with the coat removed under a Leica dissecting stereomicroscope (MZ16 FA, Germany). The samples were then subject to histochemical staining for GUS activity as previously described [33], [56]. Highly similar GUS staining results were obtained from the three reporter lines. Light microscopy was conducted under Leica dissecting microscope equipped with a digital camera.

### Plasmalemma H^+^-ATPase assay

The seeds of WT, *hrs1-1* and CP lines were sown on 1/2 MS plates, followed by cold stratification and germination as indicated above. Samples were collected at 0, 24, and 48 HOG. The preparation of plasma membrane (PM) fraction for plasmalemma H^+^-ATPase assay was conducted as reported previously [57], [58]. A protein assay kit (Bio-Rad Inc., USA) was used to determine the protein content in PM fraction. All steps were performed at 4°C. The plasmalemma H^+^-ATPase activity was assayed following published work, with the level of ATPase activity measured by monitoring the oxidation of NADH coupled to ATP hydrolysis [43]. The assay was repeated three times using independently collected seed samples, with similar results obtained among the three repeats.

### Statistical analysis

Statistical analysis of experimental data (presented as means ± SD) was conducted using ANOVA with the SPSS software package (Chicago, IL, USA).

## Supporting Information

Figure S1 Comparisons of the germination time courses of wild type (WT) control, four single mutants (*hrs1-1*, *abi3-8*, *abi4-1* and *abi5-7*), and three double mutants (*hrs1-1abi3-8*, *hrs1-1abi4-1* and *hrs1-1abi5-7*) on 1/2 MS medium. The percentages of radicle emergence (means ± SD, each calculated using the results from triplicate samples) of the eight genotypes were recorded daily for six days. The dataset displayed is typical of four independent germination assays.
(TIF)

Figure S2 Comparisons of the deduced amino acid sequences of HRS1 and its sequence homologs in *Populus trichocarpa* (XP_002311443), *Ricinus communis* (XP_002529155), *Vitis vinifera* (XP_002281762), *Zea mays* (NP_001136626), *Sorghum bicolor* (XP_002441840), and *Oryza sativa* ssp. *japonica* (NP_001046702, encoded by *Os02g0325600*). The sequence homologs of HRS1 were found in many plant species by BLASTP search in the NCBI (http://www.ncbi.nlm.nih.gov/genbank/) database. The six homologs shown were selected as representative of those found in monocotyledonous and dicotyledonous plants. The EAR-like motif is colored in red. The identities among the compared sequences range from 40% to 50%. Conserved residues are indicated by the asterisks, whereas semi-conserved and conserved substitutions are marked by the single and double dot symbols, respectively.
(TIF)

Table S1 Oligonucleotide primers used in this work.
(PDF)
