# Supplementary figures and images for: HRS1 Acts as a Negative Regulator of Abscisic Acid Signaling to Promote Timely Germination of Arabidopsis Seeds
Source: PLoS One. 2012 Apr 24;7(4):e35764. doi: 10.1371/journal.pone.0035764 (PMC3335793; doi:10.1371/journal.pone.0035764)

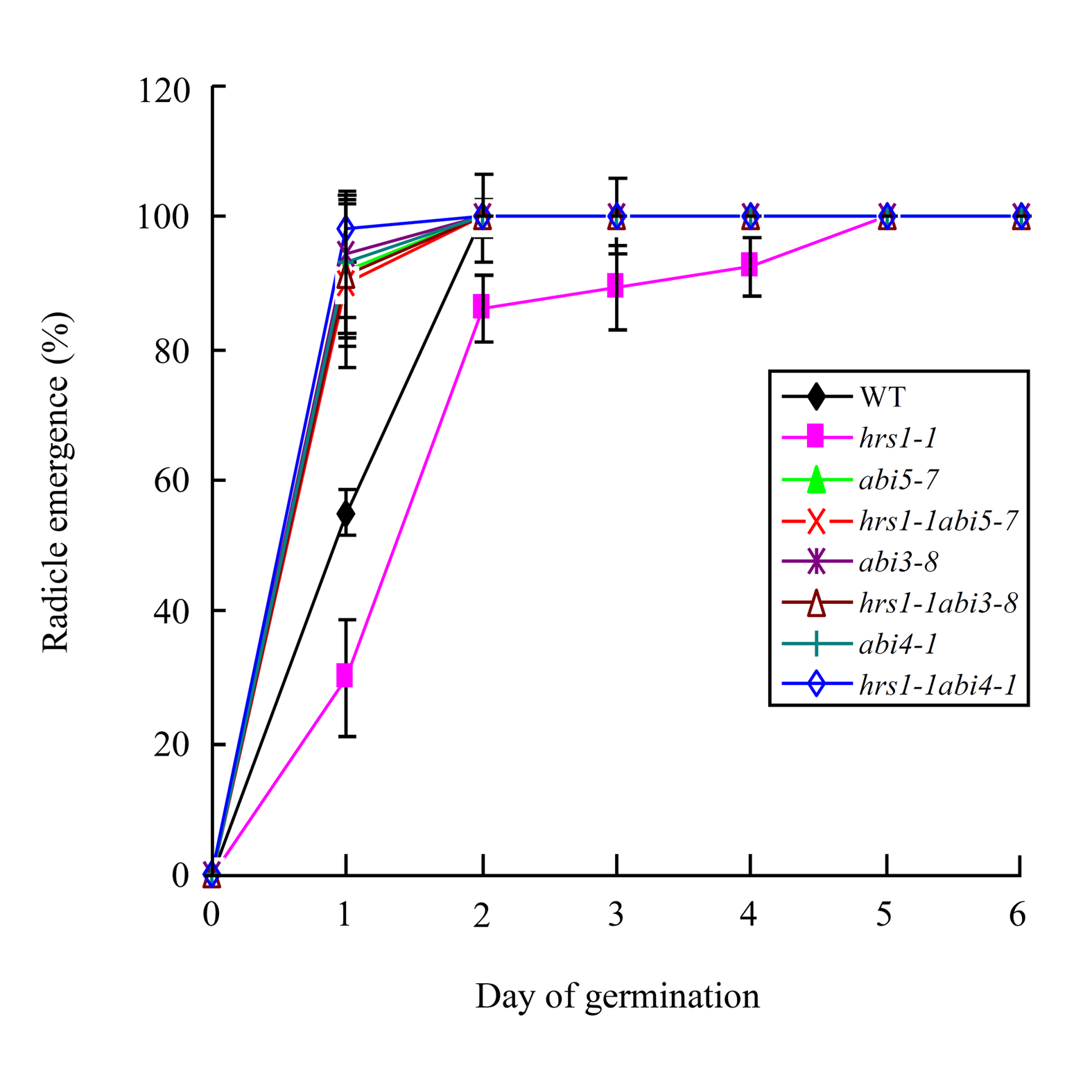

Supplement: Figure S1 — Comparisons of the germination time courses of wild type (WT) control, four single mutants (hrs1-1, abi3-8, abi4-1 and abi5-7), and three double mutants (hrs1-1abi3-8, hrs1-1abi4-1 and hrs1-1abi5-7) on 1/2 MS medium. The percentages of radicle emergence (means ± SD, each calculated using the results from triplicate samples) of the eight genotypes were recorded daily for six days. The dataset displayed is typical of four independent germination assays. (TIF) [file pone.0035764.s001.tif]

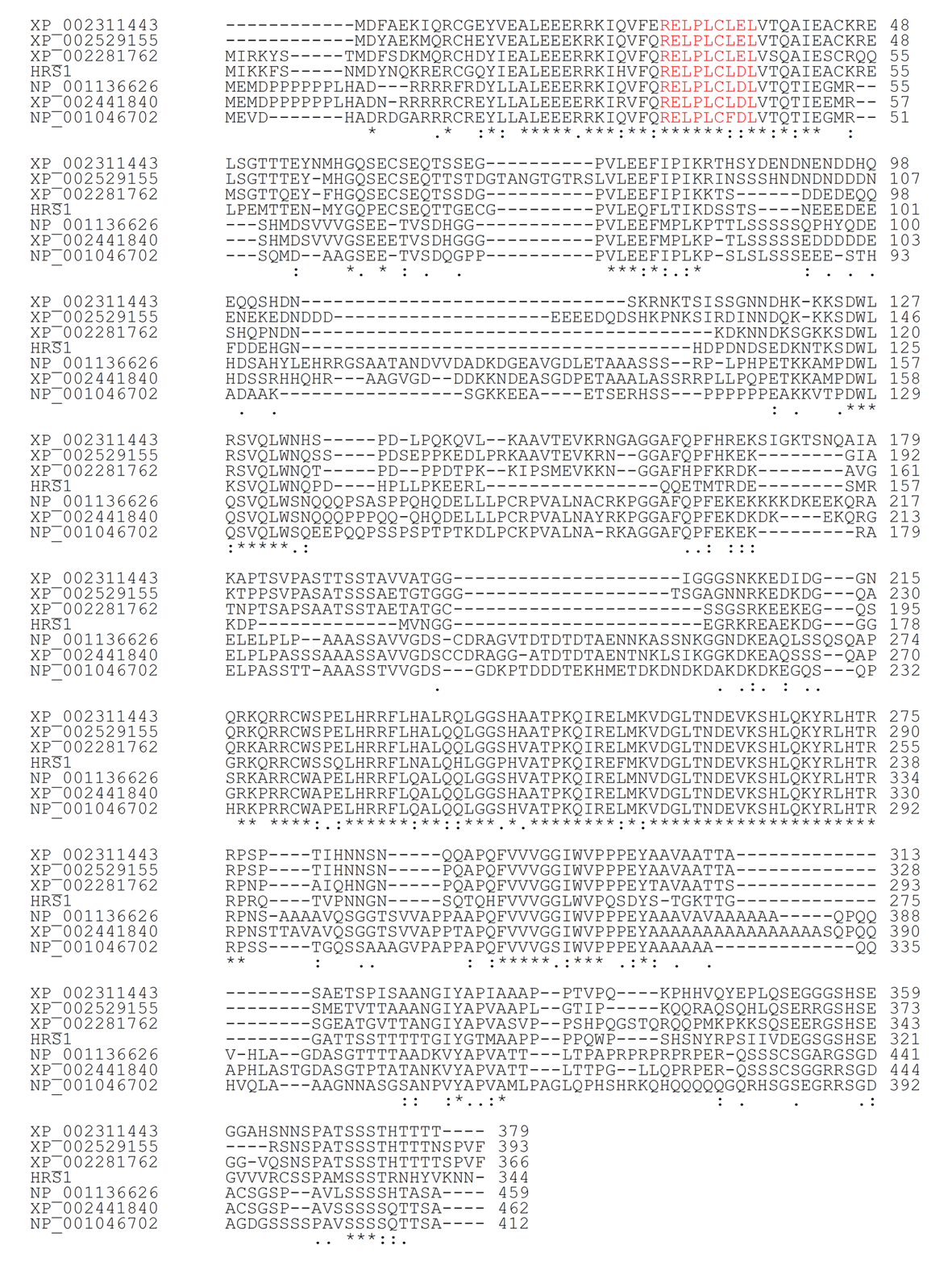

Supplement: Figure S2 — Comparisons of the deduced amino acid sequences of HRS1 and its sequence homologs in Populus trichocarpa (XP_002311443), Ricinus communis (XP_002529155), Vitis vinifera (XP_002281762), Zea mays (NP_001136626), Sorghum bicolor (XP_002441840), and Oryza sativa ssp. japonica (NP_001046702, encoded by Os02g0325600). The sequence homologs of HRS1 were found in many plant species by BLASTP search in the NCBI (http://www.ncbi.nlm.nih.gov/genbank/) database. The six homologs shown were selected as representative of those found in monocotyledonous and dicotyledonous plants. The EAR-like motif is colored in red. The identities among the compared sequences range from 40% to 50%. Conserved residues are indicated by the asterisks, whereas semi-conserved and conserved substitutions are marked by the single and double dot symbols, respectively. (TIF) [file pone.0035764.s002.tif]
