# Supplementary material for: HRS1 Acts as a Negative Regulator of Abscisic Acid Signaling to Promote Timely Germination of Arabidopsis Seeds
Source: PLoS One. 2012 Apr 24;7(4):e35764. doi: 10.1371/journal.pone.0035764 (PMC3335793; doi:10.1371/journal.pone.0035764)
Supplement: Table S1 — Oligonucleotide primers used in this work. (PDF) [file pone.0035764.s003.pdf]

Table S1. Oligonucleotide primers used in this work.

Primer	Sequence (5′-3′)	Use
abi1-2-LP<br>abi1-2-RP	AGGAAACCCTTATTGAAATTC<br>CTCTGTTCTGCTGATCATCT	Identification of *abi1-2* allele (use with LBa1)
hab1-1-LP<br>hab1-1-RP	ACAATGGCTTGTAGGTTGCTG<br>GCTTCAGGTTCTGGTCTTGAAC	Identification of *hab1-1* allele (use with LBa1)
LBa1	TGGTTCACGTAGTGGGCCATCG	Identification of *abi1-2* and *hab1-1*
abi3-F<br>abi3-R	GCGATGAAGCAGCTTTTAAAGCGAATT<br>GGTGGAGGAACCCATGTTGAAG	Identification of *abi3-8* by dCAPS marker. The *EcoR*I digestion products are either 180 bp + 20 bp (*abi3-8*) or 200 bp (wild type allele)
abi4-F<br>abi4-R	GTCACGTGCTCAGCTCAACTT<br>TTAAAAGGGATACCGTACGGAC	Identification of *abi4-1* by dCAPS marker. The *NIaI*V digestion products are either 191 bp (*abi4-1*) or 161 bp + 30 bp (wild type allele)
abi5-F<br>abi5-R	GAGTCGTCCATGGCGCAAG<br>GATTGTTATTATTCTCCTCTGGGAT	Identification of *abi5-7* by dCAPS marker. The *BamH*I digestion products are either 207 bp (*abi5-7*) or 187 bp + 20 bp (wild type allele)
pHRS1-F<br>pHRS1-R	AAT<u>GAGCTC</u>AGCTAGTTTTCGAGTTCAAGCTAG<br>ATT<u>AAGCTT</u>GATGATACTTTAGGGACTTAATTT	Amplifying the 5´ flanking sequence of *HRS1* (1617 bp) for preparing complementation and *pHRS1::HRS1-GFP* lines
HRS1-F1<br>HRS1-R1	TTA<u>AAGCTT</u>ATGATTAAAAAGTTCAGC<br>TTA<u>GGATCC</u>TTAATTATTCTTGACGTA	Amplifying the genomic sequence of *HRS1* (1615 bp) for preparing complementation lines
HRS1-F2<br>HRS1-R2	TTA<u>AAGCTT</u>ATGATTAAAAAGTTCAGC<br>TAA<u>GTCGAC</u>CATTATTCTTGACGTAATG	Amplifying the cDNA sequence of *HRS1* (1032 bp) for constructing *pHRS1::HRS1-GFP* transgenic lines
HRS1-F3<br>HRS1-R3	AACATTTCGTAGTCGTCGGTG<br>ACGGGTAGAAGAAGACATCGC	Specific for *HRS1* transcripts, used in quantitative PCR assay
ABI3-F<br>ABI3-R	ACAGGGATGGAAACCAGAAAA<br>TTCCAAACACGAGAGGTTCC	Specific for *ABI3* transcripts, used in quantitative PCR assay
ABI4-F<br>ABI4-R	AGGGACAATTCCAACACCAAC<br>CCACTTCCTCCTTGTTCCTG	Specific for *ABI4* transcripts, used in quantitative PCR assay
ABI5-F<br>ABI5-R	AACCTAATCCAAACCCGAACC<br>TACCCTCCTCCTCCTGTCCT	Specific for *ABI5* transcripts, used in quantitative PCR assay
ACT8-F<br>ACT8-R	ATATGCCTATCTACGAGGGTTTC<br>ATACAATTTCCCGTTCTGCTGT	Specific for *ACT8* transcripts, used in quantitative PCR assay as internal standard

The underlined nucleotides form *Sac*I (GAGCTC), *Hind*III (AAGCTT), *BamH*I (GGATCC) or *Sal*I (GTCGAC) restriction digestion sites.
